# Carney Complex: A Rare Case of Multicentric Cardiac Myxoma Associated with Endocrinopathy

**DOI:** 10.1155/2018/2959041

**Published:** 2018-07-02

**Authors:** Yehia Saleh, Basma Hammad, Abdallah Almaghraby, Ola Abdelkarim, Mohamed Seleem, Mahmoud Abdelnaby, Hoda Shehata, Mahmoud Hammad, Bassem Ramadan, Mohamed Elshafei, Eman Elsharkawy, Mohamed Ayman Abdel-hay

**Affiliations:** ^1^Michigan State University, East Lansing, MI, USA; ^2^Faculty of Medicine, Alexandria University, Alexandria, Egypt; ^3^Massachusetts General Hospital and Harvard Medical School, Boston, MA, USA

## Abstract

Carney complex is a rare autosomal dominant disorder characterized by multiple tumors, including cardiac and extracardiac myxomas, skin lesions, and various endocrine disorders. We are reporting a 21-year-old female patient with past surgical history significant for excision of a cutaneous myxoma who presented with multicentric cardiac myxomas involving the four cardiac chambers. She also presented with endocrinal disorders in the form of an enlarged right lobe of the thyroid, hyperthyroid state, and an incidentally noted adrenal cyst; hence, she was diagnosed with carney complex syndrome.

## 1. Introduction

Cardiac myxoma is the most common benign cardiac tumor. It usually presents as a sporadic isolated condition in the left atrium of middle-aged women [1]. A condition of multiple cardiac myxomas was reported in the familial forms. Carney complex (CNC) is one of those familial forms characterized by multiple cardiac myxomas, skin lesions, and various endocrine disorders [2].

## 2. Case Presentation

**Figure 1:**
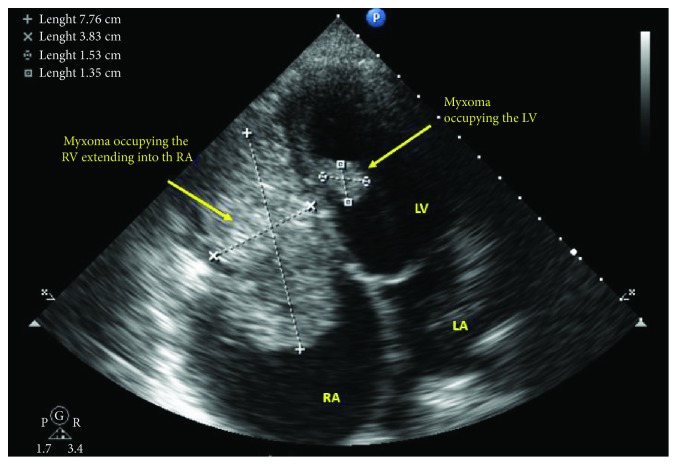
Transthoracic echocardiogram modified apical 4-chamber view showing a large mass in the right ventricle and atrium measuring 7.7 × 3.8 cm and another mass in the left ventricle attached to interventricular septum measuring 1.5 × 1.3 cm.

**Figure 2:**
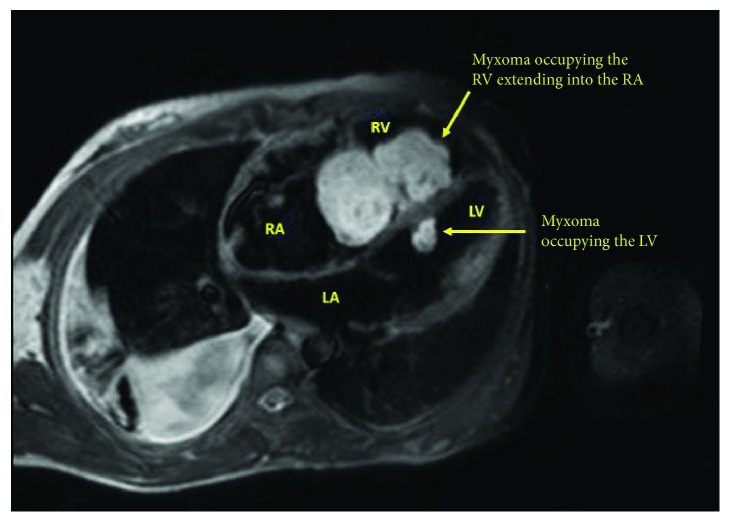
Cardiac magnetic resonance showing a myxoma occupying the right ventricle and atrium and another myxoma in the left ventricle attached to the interventricular septum.

**Figure 3:**
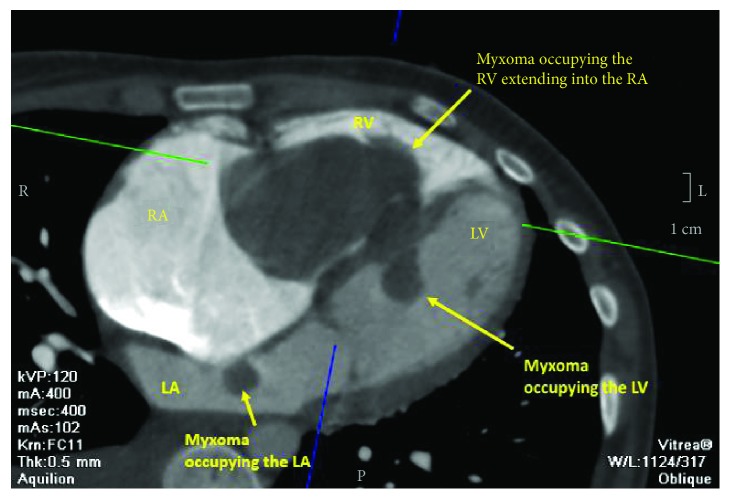
Computed tomography showing a large myxoma occupying the right ventricle and atrium, another myxoma occupying the left atrium, and a third myxoma occupying the left ventricle.

**Figure 4:**
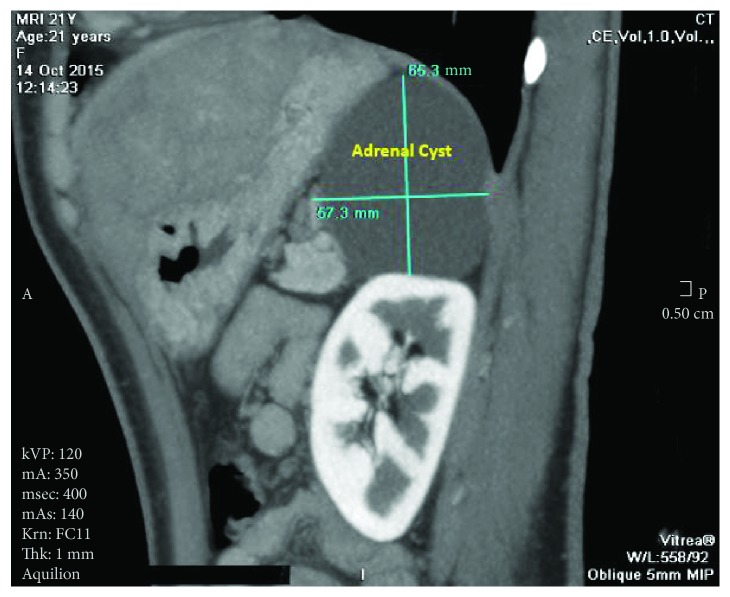
Computed tomography showing a large adrenal cyst measuring 65 × 57 mm.

A 21-year-old female patient presented with progressive exertional dyspnea and irregular palpitations for 3 months. She had past surgical history significant for excision of a cutaneous myxoma in her left arm. Physical examination revealed a high jugular venous pressure and a diastolic murmur. An electrocardiogram showed atrial fibrillation. Laboratory investigations were within normal limits except for a low TSH and elevated free T3 and T4. Transthoracic echocardiography (TTE) showed a large echogenic mobile mass with central constriction attached to the interventricular septum (IVS), occupying the entire right atrium and right ventricle (RV) and obstructing the flow of the tricuspid valve. There were two other masses of the same echogenicity: one was occupying the left ventricle (LV) and the other was in the left atrium attached to the interatrial septum at the site of fossa ovalis (Figure 1, Videos 1–5). The left ventricular dimensions and function were normal. Cardiac magnetic resonance showed similar findings with no septal invasion and tissue characterization suggestive of multiple myxomas (Figure 2). Computed tomography of the chest, abdomen, and pelvis revealed the same findings (Figure 3) in addition to an enlarged thyroid nodule and a left adrenal cyst that measures 65 × 57 mm (Figure 4). Ultrasonography of the thyroid gland revealed a markedly enlarged right lobe of the thyroid with normal vascularity. Serum aldosterone, dexamethasone suppression test, dehydroepiandrosterone sulfate, and 24-hour urine metanephrines were within normal limits.

**Figure 5:**
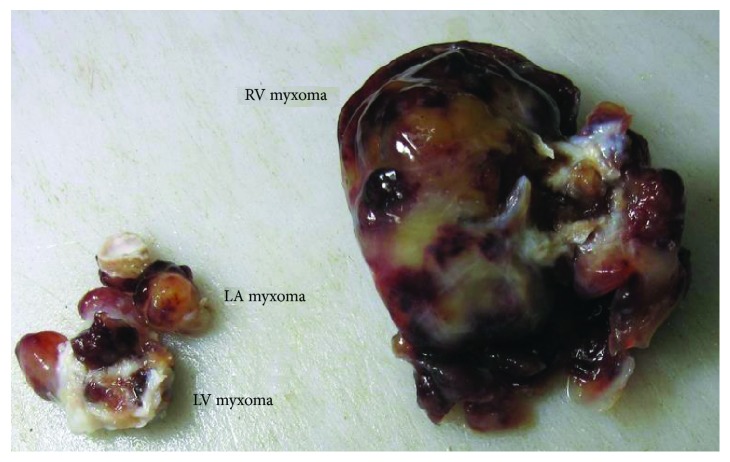
Gross specimen of the myxomas.

**Figure 6:**
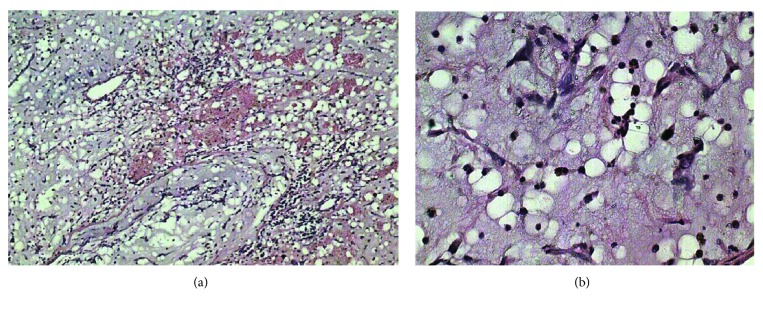
(a, b) Histopathology of mass showing myxomatous cellular proliferations with sparse collagen fibers consistent with myxoma.

**Figure 7:**
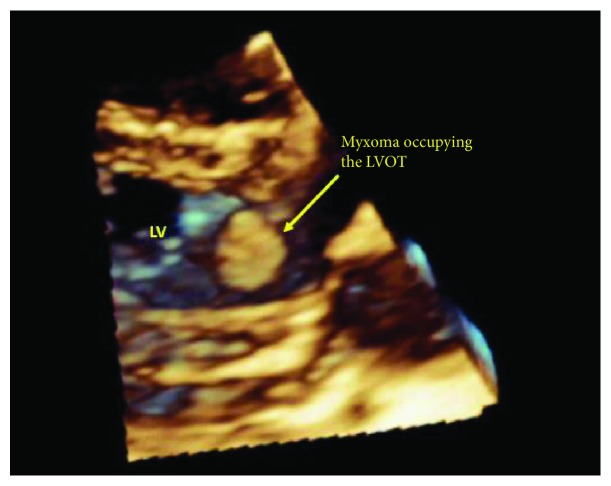
Transthoracic echocardiogram parasternal long-axis view 3-dimensional reconstruction showing a myxoma in the left ventricular outflow tract.

**Figure 8:**
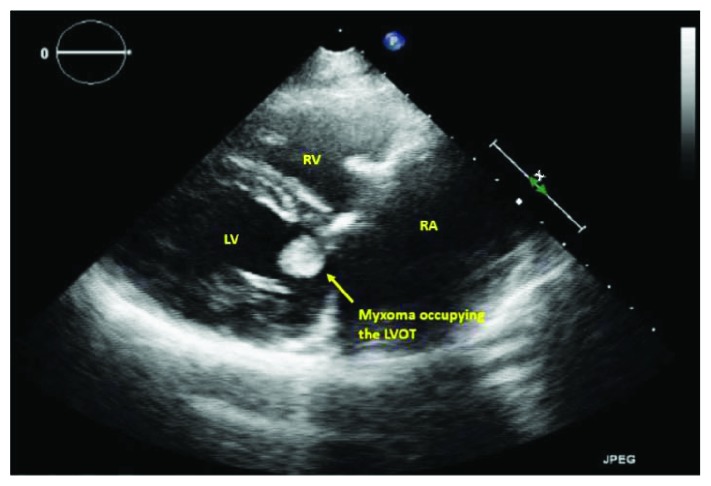
Transthoracic echocardiogram modified parasternal long-axis view showing a myxoma in the left ventricular outflow tract.

The patient underwent surgery where all three masses were excised. However, the tricuspid valve was inseparable from the RV mass; hence, it was replaced with a tissue prosthesis. The masses were grossly reddish grey in color, fleshy, and gelatinous in consistency (Figure 5). The histopathological examination of the excised masses revealed myxomatous cellular proliferations with sparse collagen fibers consistent with multiple myxomas (Figures 6(a) and 6(b)). The patient was followed up on 6-month intervals. After 2 years of follow-up, the adrenal cyst was stable in size; however, TTE showed a mass in the left ventricular outflow tract (Figures 7 and 8, Videos 6–10). The newly developed mass was surgically excised, and histopathology revealed myxomatous tissue.

## 3. Discussion

Primary intracardiac tumors are extremely rare in comparison to secondary tumors. However, myxoma is the most common primary cardiac neoplasm [1]. Cardiac myxoma usually presents as a sporadic mass; it can occur in any chamber of the heart but mainly occurs in the left atrium, and it affects women more than men with a mean age of 56. If myxoma presents as multiple masses, which rarely occurs, it is usually a familial form [2].

Carney complex (CNC) is one of the familial forms inherited in an autosomal dominant fashion. CNC is most frequently associated with mutations in the protein kinase A type I-alpha regulatory subunit gene (*PRKAR1A*). However, twenty-five percent of the cases occur sporadically secondary to de novo mutation [3]. CNC is characterized by multiple tumors, including cardiac and extracardiac myxomas, skin lesions, and various endocrine disorders [2].

Cardiac myxomas associated with CNC are characterized by the following: occurring at a younger age, multicentric, and a higher tendency of recurrence after resection in comparison to solitary ones [2, 4]. Our patient presented with three myxomas initially and had a recurrence after 2 years in a different location.

CNC-associated skin lesions can present as a spotty skin pigmentation which occurs in more than 80% of patients [5] or a cutaneous myxoma which is a benign dermal tumor present in less than 50% of patients. Cutaneous myxoma is a well-demarcated subcutaneous nodule that can reach 15 mm in diameter and most commonly affects the head, neck, and trunk. When nodules are confirmed to be myxomas histologically, the diagnosis of CNC is highly suggested [5, 6]. Hence, we would suggest screening any patient who present with cutaneous myxomas for other features of CNC. Our patient did not develop any skin pigmentation. However, she had history of a surgically removed cutaneous myxoma.

Endocrine abnormalities due to tumors of the adrenal and pituitary glands or testicular tumors are the most frequent systemic manifestations of CNC. Cushing's syndrome can occur in up to 45% of patients diagnosed with CNC [7]. Our patient had no symptoms that would suggest Cushing's syndrome, and the adrenal gland laboratory work-up was within normal limits. However, an adrenal cyst was detected on computed tomography and was followed up for 2 years without any change in size.

Thyroid disease is also common; almost 75% of patients with CNC will develop thyroid nodules typically early in life, and 25% will develop adenomatous disease. Less than 10% will develop thyroid cancer. Hence, fine-needle aspiration is indicated for patients with thyroid nodules. Regardless of the previous pathologies, most CNC patients are euthyroid [8]. Our patient presented with a nodule and was diagnosed with hyperthyroidism which is uncommon.

In order to diagnose CNC, Stratakis et al. defined the diagnostic criteria (Table 1). Diagnosis is established if the patient has two or more major criteria, or, alternatively, one major criterion in addition to either an inactivating mutation of the *PRKAR1A* gene or a first-degree relative has CNC [9, 10].

Although the clinical diagnostic criteria are reliable, nowadays genetic testing for mutations in the *PRKAR1A* gene is more commonly used for diagnostic certainty. In addition, genetic screening of potentially affected family members of patients with CNC could be useful especially in younger family members. However, clinical surveillance is still advisable for family members even when a *PRKAR1A* pathogenic variant is not identified.

In cases of cardiac myxomas, surgical resection is the treatment of choice with a 6-month follow-up via TTE afterwards due to the high incidence of recurrence. In addition, a multidisciplinary approach is recommended in order to screen for endocrinopathies and malignancies. Clinical surveillance is advisable for family members and possibly genetic testing. In our patient, clinical screening of the family was not significant; hence, we concluded she developed a de novo mutation.

## 4. Conclusion

Multiple intracardiac myxomas account for less than 5% of all cases of myxoma. Its presence should warrant screening for endocrinal disorders and should be treated by complete surgical excision with a generous safety margin in addition to close follow-up as it carries high risk of recurrence.

## Figures and Tables

**Table 1:** Diagnostic criteria for carney complex.

(1) Spotty skin pigmentation with a typical distribution (lips, conjunctiva and inner or outer canthi, vaginal and penile mucosa)
(2) Myxoma (cutaneous and mucosal)^a^
(3) Cardiac myxoma^a^
(4) Breast myxomatosis^a^ or fat-suppressed magnetic resonance imaging findings suggestive of this diagnosis
(5) PPNAD^a^ or paradoxical positive response of urinary glucocorticosteroids to dexamethasone administration during Liddle's test
(6) Acromegaly due to GH-producing adenoma^a^
(7) LCCSCT^a^ or characteristic calcification on testicular ultrasonography
(8) Thyroid carcinoma^a^ or multiple, hypoechoic nodules on thyroid ultrasonography, in a young patient
(9) Psammomatous melanotic schwannoma^a^
(10) Blue nevus, epithelioid blue nevus (multiple)^a^
(11) Breast ductal adenoma (multiple)^a^
(12) Osteochondromyxoma^a^
*Supplemental criteria*
(1) Affected first-degree relative
(2) Inactivating mutation of the *PRKARIA* gene

To make a diagnostic of CNC, a patient must either (1) exhibit two of the manifestations of the diseases listed or (2) exhibit one of these manifestations and meet one of the supplemental criteria (an affected first-degree relative or an inactivating mutation of the PRKARIA gene). ^a^With histologic information.

## Supplementary Materials

Supplementary 1 Video 1: an echocardiogram in parasternal short-axis view showing a dilated right ventricle containing a large highly mobile myxoma.

Supplementary 2 Video 2: an echocardiogram in an atypical view showing a large myxoma in the right ventricle, a myxoma in the left ventricle, and another myxoma in the left atrium.

Supplementary 3 Video 3: an echocardiogram in subcostal view showing a large myxoma in the right ventricle, dilated inferior vena cava, and a localized pericardial effusion.

Supplementary 4 Video 4: an echocardiogram in tricuspid inflow view showing a large myxoma prolapsing into a dilated right atrium.

Supplementary 5 Video 5: an echocardiogram in apical four-chamber view showing a large myxoma in the right ventricle (causing obstruction of the blood flow across the tricuspid valve), a myxoma in the left ventricle, and another myxoma in the left atrium.

Supplementary 6 Video 6: an echocardiogram in a modified tricuspid inflow view showing the tricuspid bioprosthesis and a myxoma occupying the left ventricular outflow tract.

Supplementary 7 Video 7: an echocardiogram in parasternal short-axis view showing a myxoma occupying the left ventricular outflow tract.

Supplementary 8 Video 8: three-dimensional reconstruction of an echocardiogram showing a myxoma occupying the left ventricular outflow tract.

Supplementary 9 Video 9: an echocardiogram in apical four-chamber view showing a myxoma occupying the left ventricular outflow tract.

Supplementary 10 Video 10: an echocardiogram in biplane view showing a myxoma occupying the left ventricular outflow tract.

## References

[1] Azevedo O., Almeida J., Nolasco T. (2010). Massive right atrial myxoma presenting as syncope and exertional dyspnea: case report. *Cardiovascular Ultrasound*.

[2] Vidaillet H. J., Seward J. B., Fyke F. E., Su W. P., Tajik A. J. (1987). “Syndrome myxoma”: a subset of patients with cardiac myxoma associated with pigmented skin lesions and peripheral and endocrine neoplasms. *Heart*.

[3] Kirschner L. S., Carney J. A., Pack S. D. (2000). Mutations of the gene encoding the protein kinase A type I-alpha regulatory subunit in patients with the carney complex. *Nature Genetics*.

[4] Irani A. D., Estrera A. L., Buja L. M., Safi H. J. (2008). Biatrial myxoma: a case report and review of the literature. *Journal of Cardiac Surgery*.

[5] Horvath A., Stratakis C. A. (2009). Carney complex and lentiginosis. *Pigment Cell & Melanoma Research*.

[6] Mateus C., Palangié A., Franck N. (2008). Heterogeneity of skin manifestations in patients with carney complex. *Journal of the American Academy of Dermatology*.

[7] Reynen K. (1996). Frequency of primary tumors of the heart. *The American Journal of Cardiology*.

[8] Stratakis C. A. (2016). Hereditary syndromes predisposing to endocrine tumors and their skin manifestations. *Reviews in Endocrine and Metabolic Disorders*.

[9] Almeida M. Q., Stratakis C. A. (2010). Carney complex and other conditions associated with micronodular adrenal hyperplasias. *Best Practice & Research Clinical Endocrinology & Metabolism*.

[10] Stratakis C. A., Kirschner L. S., Carney J. A. (2001). Clinical and molecular features of the carney complex: diagnostic criteria and recommendations for patient evaluation. *The Journal of Clinical Endocrinology & Metabolism*.

